# Cross-cultural validation of the Chinese version of the lean management scale for nursing services in hospitals

**DOI:** 10.3389/fpubh.2026.1895347

**Published:** 2026-07-29

**Authors:** Xue Du, Liyun Tang, Lei Tang

**Affiliations:** 1 Department of Hematology, First Affiliated Hospital of Jinzhou Medical University, Jinzhou, China; 2 Department of Nursing, First Affiliated Hospital of Jinzhou Medical University, Jinzhou, China; 3 Department of Pediatrics, Jinzhou Women's and Children's Hospital, Jinzhou, China

**Keywords:** lean management, nursing management, nursing services, psychometrics, reliability, validity

## Abstract

**Background:**

Lean management has been increasingly applied in nursing services to improve workflow efficiency and reduce waste in care processes. This study aimed to translate, culturally adapt, and evaluate the psychometric properties of the Lean Management Scale for Nursing Services in Hospitals (LMS-N) among Chinese clinical nurses.

**Methods:**

A total of 2,127 clinical nurses were recruited from four tertiary hospitals in Liaoning Province, China, using convenience sampling. Following Brislin’s translation guidelines, the LMS-N was translated and culturally adapted into Chinese. Psychometric evaluation included item analysis, internal consistency and test–retest reliability, exploratory and confirmatory factor analyses, parallel analysis, convergent and discriminant validity, and measurement invariance testing.

**Results:**

Parallel analysis and exploratory factor analysis supported a five-factor structure, explaining 62.609% of the total variance. Confirmatory factor analysis demonstrated acceptable model fit (CMIN/DF = 2.130, SRMR = 0.046, RMSEA = 0.032, GFI = 0.967, AGFI = 0.958, NFI = 0.971, IFI = 0.985, RFI = 0.967, CFI = 0.985). Reliability indices indicated satisfactory internal consistency (Cronbach’s *α* = 0.933; McDonald’s *ω* = 0.931) and test–retest reliability (0.880). Measurement invariance across gender, department, and educational attainment was supported.

**Conclusion:**

The Chinese version of the LMS-N demonstrated acceptable reliability and validity among clinical nurses from tertiary hospitals in one province of China. The scale may be useful for assessing lean management implementation in nursing practice, although further validation in more diverse settings is warranted.

## Introduction

Amid growing tensions between the supply and demand of healthcare resources and increasing expectations for high-quality patient care (1), achieving efficient and high-standard nursing services through scientific management approaches has become a pressing challenge faced by healthcare systems worldwide (2). Traditional nursing management models often suffer from redundant processes, resource waste, and low efficiency, which not only hinder the continuous improvement of service quality but also contribute to excessive workloads and burnout among healthcare professionals (3). In response, the concept of Lean Management—originating from the manufacturing industry—has been increasingly adopted in healthcare (4). Its core principles involve reducing non–value-added activities and process variability, improving working conditions, and continuously optimizing workflows through performance monitoring (5). Emerging evidence suggests that lean management exerts a positive influence on nursing services by identifying and eliminating waste in care delivery (e.g., time and resource inefficiencies) and enhancing service efficiency through process standardization (6). Moreover, it promotes order within nursing units and fosters a standardized, safer, and more supportive working environment (7). Lean practices have already shown significant improvements in areas such as operating room turnover, hospital-acquired infection reduction, and patient experience (8).

In China, hospitals face unique challenges, including high patient volumes, limited nursing resources, and strong expectations for efficiency under government-driven healthcare reforms (9). These contextual factors make the adaptation of lean principles to nursing services both timely and necessary. Our study was conducted in Liaoning Province, an economically diverse region in Northeast China where tertiary hospitals are under pressure to balance service quality with resource constraints (10). By situating the study in this region, we aimed to test whether lean nursing management practices could be measured reliably in a real-world context representative of Chinese hospital systems.

As one of the most patient-facing and process-intensive components of hospital operations, nursing services play a critical role in determining healthcare quality and patient safety (11). However, compared to more standardized domains such as equipment management and pharmaceutical supply, the lean transformation of nursing services faces unique challenges. On one hand, the dynamic nature and human-centered attributes of nursing work make it difficult to apply manufacturing-based standard evaluation metrics (12). On the other hand, existing studies often rely on semi-structured questionnaires or scales focused on stress and organizational factors to evaluate lean management outcomes, with few tools specifically designed to measure the level of lean implementation in nursing services. Many of the current instruments are adapted from industrial settings (13), while those in healthcare either fail to focus on lean thinking within nursing services or emphasize only lean leadership (14). One notable exception is a hospital lean improvement scale for nurses comprising 79 items across 12 dimensions (15), which poses practical limitations due to its length and complexity. Recently, Turkish scholar Çiğdem et al. developed the Lean Management in Nursing Services in Hospitals (LMS-N) scale based on core lean principles in 2023 (5). This 22-item, 5-dimension instrument demonstrated strong reliability and validity through empirical testing, filling a notable gap in the assessment of lean management in nursing. However, in China, there is currently no localized, nursing-specific lean management assessment tool. Existing research still relies heavily on generic quality management instruments, which often lack cultural adaptation and relevance to nursing-specific contexts.

LMS-N conceptualizes lean practices across five key factors: Management Support, Visual Stock Management, Work Environment Layout, Preventive Reporting System, and Waste Detection (5). Each of these reflects not only general lean principles but also their specific relevance to Chinese nursing practice (16). For example, Management Support is crucial in hierarchical hospital systems where administrative endorsement directly influences nursing workflow reform. Visual Stock Management addresses the chronic issue of supply shortages and inventory inefficiencies that nurses frequently encounter. Work Environment Layout focuses on optimizing the physical workspace to minimize unnecessary movement and enhance workflow efficiency—a critical concern in China’s often overcrowded nursing stations and patient units. Preventive Reporting System emphasizes the proactive identification and reporting of near-miss events and potential errors, aligning with national patient safety initiatives and shifting nursing culture toward transparency and continuous learning. Finally, Waste Detection captures nurses’ ability to identify and eliminate non-value-added activities, which is essential for improving efficiency in high-volume hospital settings.

Recent literature further underscores both the growing breadth of lean management research in healthcare and the persistent absence of nursing-specific, China-validated instruments. A 2025 systematic review synthesizing 60 studies found that Lean implementation in hospitals has produced measurable improvements across efficiency, quality, cost, and patient/staff satisfaction, yet noted that no prior study had quantitatively synthesized these effects together, highlighting that the evidence base—while expanding—remains fragmented (17). Beyond nursing wards, lean principles have recently been extended to other hospital sectors, including pharmacy operations (18) and, within the Chinese healthcare reform context specifically, hospital-level adaptation to medical insurance payment reform (DRG/DIP) (19), demonstrating that lean management is an active and evolving area of inquiry in China’s health system. At the same time, instrument-validation research among Chinese nurses has flourished in adjacent domains—for example, the recent development of the Chinese Nursing Core Competence Scale (20) and the cross-cultural validation of the Nursing Time Management Scale (21) and the Digital Competence Questionnaire for clinical nurses [X6]—yet no equivalent, psychometrically validated instrument exists for assessing lean management implementation specifically within Chinese nursing services. This persistent gap, despite parallel advances in both lean healthcare research and Chinese nursing scale validation, further motivates the present study.

By explicitly linking these five dimensions to nursing services in Chinese hospitals, this study provides a theoretically grounded framework for assessing lean management practices in the local context. Our validation of the Chinese version of the LMS-N therefore offers not only a practical assessment tool for nurse managers but also empirical evidence supporting the cross-cultural applicability of lean nursing concepts. Beyond its psychometric contribution, the availability of a validated nursing-specific lean management instrument has broader implications for public health policy and healthcare governance. At the hospital level, the LMS-N may facilitate routine quality monitoring, support data-driven managerial decision-making, and help identify areas for workflow optimization and resource allocation. At the workforce level, it may assist nursing administrators in evaluating organizational environments and developing strategies to improve staff efficiency, job sustainability, and quality of care. More broadly, within the context of ongoing healthcare system reform in China, standardized assessment of lean nursing practices may contribute to strengthening hospital performance evaluation frameworks and promoting high-quality, efficient, and patient-centered healthcare delivery.

## Methods

### Study design

This was a methodological study involving translation, cross-cultural adaptation, and psychometric testing of the LMS-N. Data were collected cross-sectionally from nurses working in hospitals in Liaoning Province, China.

### Sample size

Data were collected from June 2025 to April 2026 using a cross-sectional survey design. Participants were recruited through convenience sampling from four tertiary hospitals across Jinzhou in Liaoning Province, China. Eligible nurses who met the inclusion criteria were invited to participate voluntarily during the study period. As the study did not aim to compare outcomes across hospitals or conduct hospital-level analyses, hospital identifiers were not collected during questionnaire administration. To ensure data quality, all returned questionnaires underwent screening for validity. Questionnaires with substantial missing data, duplicate submissions, or obviously invalid response patterns were excluded from the final analysis. A total of 2,250 questionnaires were distributed, and 2,127 valid responses were retained, resulting in an effective response rate of 94.53%.

In determining the psychometric properties of the scale, we followed Kendall’s recommendation that the sample size should be 5–10 participants per item (22). Using a conservative ratio of 10 participants per item and accounting for a potential 20% attrition rate, the minimum required sample size for the 22-item LMS-N scale was calculated as 275 participants. In this study, both exploratory factor analysis (EFA) and confirmatory factor analysis (CFA) were planned. According to established guidelines (23), a minimum of 150 participants is recommended for EFA and at least 200 for CFA. Ultimately, to establish independent samples for factor analysis, the full dataset was randomly divided into two approximately equal subsamples using the “Random Sample of Cases” procedure in SPSS. Sample 1 (*n* = 1,027) was used for exploratory factor analysis (EFA), and Sample 2 (*n* = 1,100) was used for confirmatory factor analysis (CFA). Comparisons of key sociodemographic characteristics indicated no significant differences between the two subsamples (Supplementary material 1).

### Participant

Inclusion criteria were as follows: (1) age ≥ 18 years; (2) possession of a registered nursing license at four tertiary hospitals; and (3) voluntary participation with full understanding of the study’s objectives and procedures. Exclusion criteria included: (1) nurses who were on leave during the survey period; and (2) those who were interns or visiting nurses at the hospital.

### Research tools

#### Sociodemographic data questionnaire

A self-designed questionnaire was developed by the researchers based on an extensive review of relevant literature. It included variables such as gender, age, educational background, years of professional experience, department, professional title and marital status.

#### Chinese version of the lean management scale for nursing services in hospitals

This scale comprised 22 items across five dimensions: Management Support (items1–8), Visual Stock Management (items 9–12), Work Environment Layout (items 13–16), Preventive Reporting System (items 17–19), and Waste Detection (items 20–22). Items 20–22 were negatively worded and were reverse-scored prior to calculating dimension and total scores (i.e., 1 → 5, 2 → 4, 3 → 3, 4 → 2, and 5 → 1). All items were rated using a five-point Likert scale ranging from 1 (“Strongly disagree”) to 5 (“Strongly agree”). After reverse scoring, higher scores indicated higher levels of lean management implementation.

Given that the original LMS-N cut-offs (1.00–2.33 low, 2.34–3.66 moderate, 3.67–5.00 high) were based on the original authors’ conventions and have not been validated in the Chinese context, categorical labels are not reported. Scores are therefore treated as continuous variables, and categorical interpretation for decision-making is discouraged until normative data are established.

### Brislin’s translation model steps

The research team obtained permission to use the original LMS-N scale by contacting its author via email and receiving formal authorization. The translation process strictly followed Brislin’s model and included the following steps (24):

1) Forward Translation: Two nursing master’s students—native Chinese speakers who had passed the College English Test Band 6 (CET-6)—independently translated the original English version into Chinese, resulting in two preliminary versions (L1 and L2). A nursing PhD with overseas academic experience then reviewed, compared, and synthesized the two versions to produce a reconciled Chinese version (L).
2) Back Translation: One university English teacher and one nursing faculty member with overseas experience—both unfamiliar with the original scale—independently back-translated version L into English, generating versions BL1 and BL2. The research team compared and analyzed the back-translated versions with the original English scale, focusing on conceptual and semantic equivalence. Items with significant discrepancies were retranslated and re-back-translated, resulting in a revised English version (BL) and corresponding Chinese version. The revised English back-translation (BL) was sent to the original author for review, and adjustments were made according to the author’s feedback. This process yielded a preliminary Chinese version of LMS-N.
3) Cultural Adaptation: The Delphi method was employed for cultural adaptation. Seven experts participated in two rounds of consultation (Table 1). They evaluated the content, semantics, phrasing, and cultural relevance of each item, suggesting refinements where necessary. A four-point Likert rating scale was used for the assessment. Based on the relevance of each item to the construct being measured, items were rated as not relevant (1 point), weakly relevant (2 points), quite relevant (3 points), or highly relevant (4 points). Based on their input, the culturally adapted Chinese version of LMS-N was finalized.

**Table 1:** Characteristics of the expert panel for Delphi process (*n* = 7).

Characteristic	Frequency	Percentage
Gender		
Male	2	28.57%
Female	5	71.42%
Age (years)		
30–39	1	14.29%
40–49	3	42.85%
50–59	2	28.57%
≥60	1	14.29%
Professional background		
Nursing administration	1	14.29%
Quality management/improvement	1	14.29%
Lean management consultant	1	14.29%
Nursing research/psychometrics	2	28.57%
Clinical nursing expert	2	28.57%
Years of professional experience		
10–15 years	2	28.57%
16–20 years	2	28.57%
>20 years	3	42.86%
Highest education level		
Master’s degree	4	57.14%
Doctoral degree	3	42.86%

Source: author composition based on study data.

Experts were purposively recruited based on the following inclusion criteria: (1) At least 10 years of clinical or managerial experience in Chinese tertiary hospitals. (2) Professional background in one or more of the following areas: Nursing administration or quality management; Lean management implementation in healthcare; Psychometrics or scale development; Clinical nursing practice with expertise in hospital operations. (3) Master’s degree or higher in nursing, healthcare management, or related fields. 4. Willingness to participate in two rounds of anonymous review.

4) Pilot Testing: A convenience sample of 30 nurses from the study hospital was selected based on inclusion and exclusion criteria to conduct a pilot test. Participants were asked whether the 22 items were easy to understand, and their feedback on item clarity and completion time was recorded. All participants completed the questionnaire in 3–5 min and reported no ambiguity. Therefore, all items were retained. Incorporating feedback from both experts and pilot participants, the research team finalized the Chinese version of LMS-N for psychometric testing.

### Data collection and quality control

Prior to the commencement of the formal survey, the researchers presented the study’s objectives, implementation plan, and anticipated outcomes in detail to the Director of the Nursing Department at four tertiary hospitals. The questionnaire was administered via https://www.wjx.cn/, a professional online survey platform widely used in China. The research team provided clinical nurses with a clear explanation of the study’s scope and data collection procedures. Eligible participants, screened according to predefined inclusion criteria, were instructed to access the electronic questionnaire system by scanning a unique QR code using their mobile devices. To ensure the validity and reliability of the collected data, two quality control measures were implemented. First, based on findings from the pilot study, a minimum completion time of 3 min was established; the survey system was programmed to automatically reject any submissions that did not meet this threshold. Second, a dual-verification mechanism using both device identifiers and IP addresses was employed to prevent duplicate submissions from the same terminal, thereby safeguarding the authenticity and uniqueness of the data at the technical level.

### Statistical analysis

Data were analyzed using IBM SPSS Statistics 27.0 and IBM AMOS 25.0. For continuous variables with a normal distribution, data were presented as mean ± standard deviation; for non-normally distributed data, the median and interquartile range (IQR) were used. Categorical variables were described using frequencies and percentages. Item discrimination was examined using the 27% extreme-groups method, in which participants were divided into the top 27% and bottom 27% of total LMS-N scores. Independent-samples *t*-tests were then conducted to compare item scores between the two groups. Items with statistically significant differences (*p* < 0.05) and sufficiently large *t*-values were considered to have acceptable discriminatory power. And item-total correlation (25). Reliability was assessed using Cronbach’s alpha coefficient and test–retest reliability. Content validity was evaluated through the item-level content validity index (I-CVI) and scale-level content validity index (S-CVI/Ave) (26). Structural validity was examined via EFA and CFA.

EFA was conducted using IBM SPSS Statistics 27.0 with Sample 1 (*n* = 1,027). Principal axis factoring was employed as the extraction method based on the Pearson correlation matrix. The suitability of the data for factor analysis was assessed using the Kaiser-Meyer-Olkin (KMO) measure of sampling adequacy and Bartlett’s test of sphericity. The number of factors to retain was determined by: (1) Kaiser’s criterion (eigenvalue > 1.0), (2) scree plot examination and (3) parallel analysis using raw data permutation. In the parallel analysis, observed eigenvalues were compared with the 95th percentile of eigenvalues generated from 100 randomly permuted datasets, and factors were retained when the observed eigenvalues exceeded the corresponding random eigenvalues. Direct oblimin rotation was applied to allow correlations among factors, as the five dimensions of lean management were theoretically expected to be interrelated. Items were retained if they had primary factor loadings ≥0.50 and cross-loadings <0.32. The final factor solution was evaluated based on both statistical adequacy and alignment with the theoretical framework of the original LMS-N scale.

CFA was conducted on Sample 2 using IBM AMOS 25.0 to evaluate the fit of the five-factor model derived from the EFA. Maximum likelihood (ML) estimation was employed. Although the items were measured using a 5-point Likert scale and therefore treated as ordinal, ML estimation was considered appropriate because of the large sample size (*n* > 1,000) and the relatively symmetric distribution of item responses. Previous methodological studies have suggested that under these conditions, ML estimation can provide stable and acceptable parameter estimates and model fit indices for ordinal data. Therefore, ML estimation was retained for the present analysis.

Convergent validity was assessed based on average variance extracted (AVE) and composite reliability (CR), while discriminant validity was determined by comparing the square root of the AVE for each construct with the inter-construct correlation coefficients (27). A *p*-value < 0.05 was considered statistically significant. Discriminant validity among the five factors was further examined using the Heterotrait-Monotrait ratio of correlations (HTMT). The HTMT value represents the ratio of the average heterotrait-heteromethod correlations (i.e., the average correlation of items across different factors) to the average monotrait-heteromethod correlations (i.e., the average correlation of items within the same factor). An HTMT value below 0.85 indicates satisfactory discriminant validity (28).

To examine whether the five-factor model of the Chinese version of the LMS-N was equivalent across gender, educational attainment, and department, this study further conducted multi-group confirmatory factor analyses to test measurement invariance. Following the hierarchical levels of invariance, three increasingly restrictive models were evaluated: configural invariance, metric invariance, and scalar invariance (29).

## Results

### Sociodemographic characteristics

A total of 2,127 participants were included in this study, with a mean age of 38.62 ± 9.47 years and an average of 15.62 ± 8.56 years of work experience. The sample primarily consisted of middle-aged female nurses with higher educational attainment and extensive professional experience, most of whom were affiliated with medical and surgical departments. Detailed information is presented in Table 2.

**Table 2:** Distribution of socio-demographic information of the participants (*n* = 2,127).

Variables		Frequency (*n*)	Percentage (%)
Gender	Males	638	29.99
	Females	1,489	70.01
Educational attainment	Professional training college	212	9.96
	Undergraduate	1,374	64.59
	Postgraduates	541	25.43
Department	Internal	621	29.19
	Surgery	584	27.45
	Emergency	481	22.61
	ICU	319	14.99
	Others	122	5.73
	Nurse	621	29.2
Professional title	Senior nurse	584	27.5
	Supervisor nurse	481	22.6
	Associate chief nurse	319	15.0
	Chief nurse	122	5.7
Marital status	Married	1,284	60.4
	Unmarried	718	33.8
	Others	125	5.9

NICU: intensive care unit; Source: author composition based on study data.

### Cultural adaptation results

A two-round Delphi consultation was conducted with a panel of seven experts. The response rate was 100% in both rounds. The expert authority coefficient (Cr) was 0.86, indicating a high level of expertise among the panel Inter-expert agreement was evaluated using Kendall’s coefficient of concordance (W). The W value increased from 0.318 in the first round to 0.374 in the second round, with both values reaching statistical significance (*p* < 0.01), indicating an improving and acceptable level of consensus among experts across rounds.

Experts unanimously agreed that the overall structure and content of the scale were culturally appropriate and aligned with the realities of China’s healthcare context. However, minor adjustments were recommended to enhance linguistic precision and contextual relevance. Specifically, two key suggestions were made:

First, the original scale was developed for a broad healthcare service system, in which the term “employees” was used generically. To improve semantic clarity and contextual alignment in the Chinese healthcare setting, experts recommended replacing it with “nurses.” Second, the term “stand-up meeting” in Item 7, while common in Western corporate culture, was rarely used in Chinese hospitals. Experts suggested translating it as “departmental morning meeting,” a term more familiar to Chinese clinical staff. Following in-depth discussions with the expert panel, both recommendations were accepted and implemented. No items were deleted during the cross-cultural adaptation and pilot testing phases. Only these targeted revisions were made to improve the scale’s accuracy and applicability in the Chinese context.

### Item analysis

Subsequently, the total scores of the 2,127 valid samples were ranked in descending order. The top 27% were classified as the high-score group, and the bottom 27% as the low-score group. According to the research of Ling Hui Kong (30), the kurtosis and skewness of the items are approximately normally distributed between −2 and +2. All 22 items in this study meet this criterion (Table 3). Independent-sample *t*-tests revealed that the critical ratio values ranged from 31.720 to 52.155, all exceeding the threshold of 3.000 and reaching statistical significance (*p* < 0.001) (31). In addition, the item–total correlation coefficients ranged from 0.546 to 0.733, all greater than 0.40 and statistically significant (p < 0.001) (32), as presented in Table 3.

**Table 3:** Chinese version of LMS-N scale’s s item analysis results.

Items	Threshold	Mean	SD	Skewness	Kurtosis	Item–total correlation coefficients	Critical ratio	*p*
Q1	[1,5]	3.43	1.315	−0.426	−1.013	0.684	41.219	<0.001
Q2	[1,5]	3.37	1.363	−0.355	−1.186	0.704	49.248	<0.001
Q3	[1,5]	3.32	1.361	−0.362	−1.166	0.707	46.914	<0.001
Q4	[1,5]	3.35	1.371	−0.369	−1.140	0.707	47.343	<0.001
Q5	[1,5]	3.30	1.352	−0.327	−1.163	0.733	52.155	<0.001
Q6	[1,5]	3.31	1.351	−0.316	−1.122	0.725	49.870	<0.001
Q7	[1,5]	3.35	1.325	−0.345	−1.120	0.710	47.812	<0.001
Q8	[1,5]	3.35	1.343	−0.360	−1.124	0.722	49.554	<0.001
Q9	[1,5]	3.36	1.372	−0.385	−1.131	0.616	38.538	<0.001
Q10	[1,5]	3.33	1.321	−0.343	−1.113	0.639	40.004	<0.001
Q11	[1,5]	3.37	1.338	−0.371	−1.131	0.611	37.618	<0.001
Q12	[1,5]	3.34	1.391	−0.359	−1.146	0.642	41.306	<0.001
Q13	[1,5]	3.38	1.343	−0.431	−1.024	0.604	35.308	<0.001
Q14	[1,5]	3.36	1.389	−0.381	−1.126	0.614	37.096	<0.001
Q15	[1,5]	3.39	1.354	−0.376	−1.117	0.635	39.592	<0.001
Q16	[1,5]	3.37	1.354	−0.400	−1.060	0.632	38.180	<0.001
Q17	[1,5]	3.41	1.328	−0.398	−1.050	0.558	32.371	<0.001
Q18	[1,5]	3.37	1.341	−0.371	−1.085	0.602	37.096	<0.001
Q19	[1,5]	3.39	1.347	−0.354	−1.138	0.585	34.655	<0.001
Q20	[1,5]	3.42	1.361	−0.393	−1.131	0.546	31.720	<0.001
Q21	[1,5]	3.32	1.362	−0.358	−1.138	0.603	37.881	<0.001
Q22	[1,5]	3.32	1.356	−0.384	−1.113	0.598	37.175	<0.001

Source: author composition based on study data.

### Content validity

To evaluate the content validity of the scale, seven experts in the field were invited to assess each item of the Chinese version of LMS-N. The I-CVI, defined as the proportion of experts rating an item as either 3 or 4 on a 4-point relevance scale, ranged from 0.857 to 1.00—all exceeding the commonly accepted threshold of 0.78 (33). Furthermore, the S-CVI/Ave, calculated using the average method, reached 0.961 (>0.80) (34).

### Construct validity

**Figure 1:**
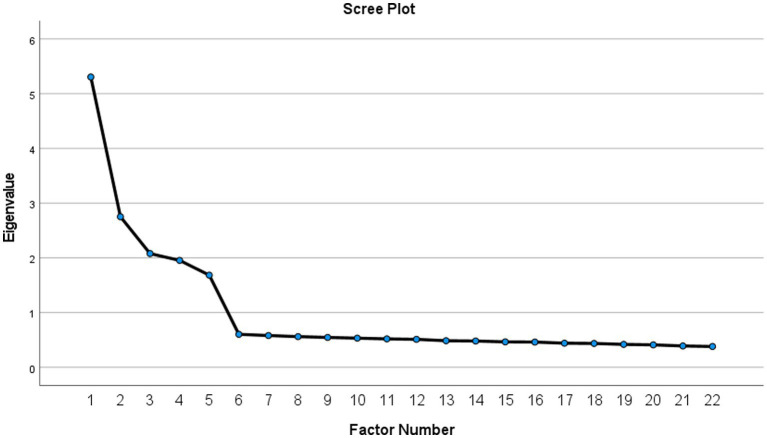
Chinese version of LMS-N five-factor scree plot (*n* = 1,027). Source: author composition based on study data.

**Figure 2:**
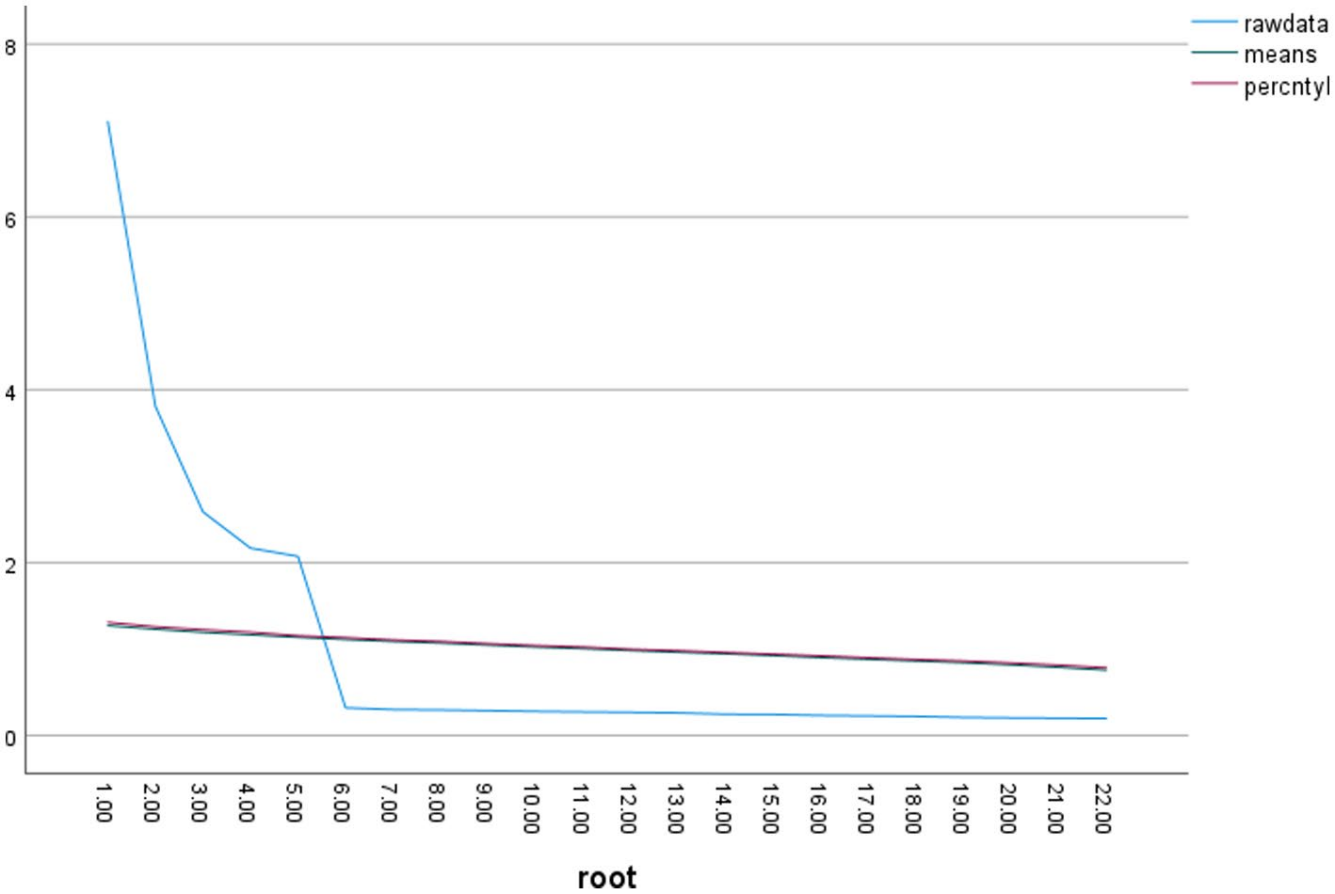
Chinese version of LMS-N parallel analysis plot (*n* = 1,027). Source: author composition based on study data.

Prior to conducting factor analysis, the suitability of Sample 1 and Sample 2 was assessed. Bartlett’s test of sphericity yielded statistically significant results for both samples, with *χ*^2^ values of 8,031.095 and 14,710.504 (df = 231, *p* < 0.001), respectively. The Kaiser-Meyer-Olkin (KMO) measures of sampling adequacy were 0.875 for Sample 1 and 0.965 for Sample 2, both exceeding the threshold of 0.80 (35), indicating that the data were appropriate for factor analysis. Principal axis factoring with direct oblimin rotation extracted five factors with eigenvalues >1.0 (Figure 1), collectively explaining 62.609% of the total variance. The scree plot supported a five-factor solution, which aligned with the theoretical structure of the original LMS-N scale. All 22 items demonstrated satisfactory factor loadings (range: 0.724–0.831), with no problematic cross-loadings (all < 0.32; Table 4). Besides, Parallel analysis demonstrated that the first five observed eigenvalues (7.10, 3.81, 2.59, 2.17, and 2.07) exceeded the corresponding 95th percentile random eigenvalues (1.31, 1.26, 1.22, 1.19, and 1.15), whereas the sixth observed eigenvalue (0.32) did not exceed the random criterion (1.13; Table 5). These findings supported retention of a five-factor solution (Figure 2).

**Table 4:** Factor loadings of the Chinese version of the LMS-N scale (*n* = 1,027).

Items	Management support	Work environment layout	Visual stock management	Waste detection	Preventive reporting system
Q8	**0.771**	0.040	0.049	0.081	0.011
Q2	**0.770**	0.064	0.007	0.055	0.043
Q5	**0.758**	0.107	0.062	0.011	0.069
Q7	**0.751**	0.028	0.065	0.035	0.049
Q4	**0.750**	0.098	0.063	0.011	0.039
Q3	**0.745**	0.011	0.054	0.067	0.054
Q6	**0.736**	0.091	0.052	0.026	0.080
Q1	**0.724**	0.064	0.077	−0.014	0.041
Q13	0.106	**0.805**	0.087	0.006	0.043
Q16	0.121	**0.794**	0.052	0.022	0.055
Q14	0.072	**0.773**	0.016	0.054	0.050
Q15	0.061	**0.762**	0.105	0.093	0.059
Q10	0.070	0.081	**0.800**	0.031	0.039
Q11	0.036	0.076	**0.793**	0.033	0.060
Q12	0.098	0.040	**0.768**	0.028	0.082
Q9	0.099	0.058	**0.768**	0.097	0.032
Q21	0.077	0.089	0.034	**0.831**	0.034
Q22	0.065	−0.012	0.048	**0.821**	0.072
Q20	0.033	0.091	0.098	**0.821**	0.090
Q19	0.064	0.065	0.080	0.040	**0.814**
Q18	0.117	0.067	0.088	0.063	**0.805**
Q17	0.073	0.060	0.033	0.090	**0.800**

Source: author composition based on study data.

**Table 5:** Parallel analysis of the Chinese version of the LMS-N scale (*n* = 1,027).

Roots	Rawdata	Mean	Percntyl
1	7.10	1.27	1.31
2	3.81	1.23	1.26
3	2.59	1.19	1.22
4	2.17	1.16	1.19
5	2.07	1.14	1.15
6	0.32	1.11	1.13
7	0.30	1.09	1.11
8	0.30	1.07	1.09
9	0.29	1.05	1.06
10	0.28	1.02	1.04
11	0.27	1.00	1.02
12	0.27	0.98	1.00
13	0.26	0.96	0.98
14	0.25	0.94	0.96
15	0.24	0.92	0.94
16	0.23	0.90	0.92
17	0.23	0.88	0.90
18	0.22	0.86	0.88
19	0.21	0.84	0.86
20	0.20	0.82	0.84
21	0.20	0.79	0.81
22	0.20	0.76	0.78

Source: author composition based on study data.

**Figure 3:**
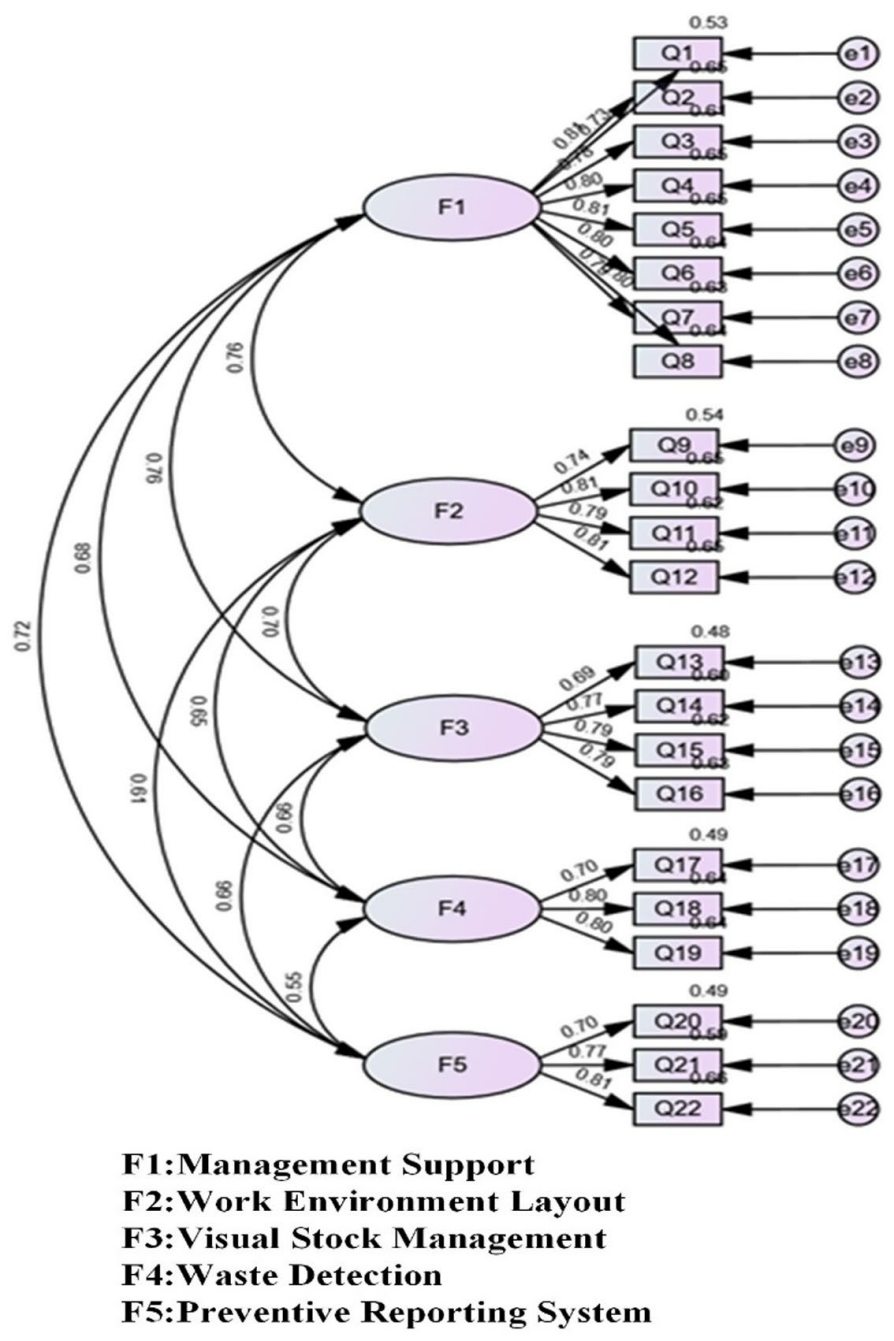
Chinese version of LMS-N standardized CFA model fit plot (*n* = 1,100). Source: author composition based on study data.

A first-order confirmatory factor model comprising five latent factors was specified. In accordance with methodological recommendations for ordinal Likert-type data, ML was employed, as it provides robust standard errors and a scaled test statistic that does not assume multivariate normality. Model fit indices indicated an acceptable fit to the data: CMIN/DF = 2.130(<3) (36), SRMR = 0.046(<0.05), RMSEA = 0.032, 90% CI [LO 90 = 0.28, HI 90 = 0.36] (<0.08) (22), AGFI = 0.958, NFI = 0.971, RFI = 0.967, GFI = 0.967, IFI = 0.985 and CFI = 0.985, all exceeding the recommended threshold of 0.90 (25). Additionally, all standardized factor loadings were above 0.50 (Figure 3), further supporting the adequacy of the model.

### Convergent and discriminant validity

Convergent validity was assessed by calculating the AVE and CR for each latent construct. The AVE values for the five dimensions were 0.626, 0.616, 0.584, 0.592, and 0.581, respectively, all exceeding the recommended threshold of 0.50 (37). Corresponding CR values were 0.930, 0.864, 0.848, 0.812, and 0.805, all above the acceptable benchmark of 0.70 (38).

To assess discriminant validity, the square roots of AVE values for each construct were calculated and found to be 0.791, 0.784, 0.764, 0.769, and 0.762, respectively (Table 6), each exceeding the inter-construct correlation coefficients (39). All HTMT values ranged from 0.536 to 0.758, well below the conservative threshold of 0.85 (Table 7).

**Table 6:** Convergent validity and discriminant validity of Chinese version of LMS-N.

Parameters of the model					Convergent validity		Discriminant validity				
Factors	Items	Standard estimate	S.E.	P	AVE	CR	F1	F2	F3	F4	F5
F1	Q1	0.730			0.626	0.930	**0.791**				
	Q2	0.806	0.046	***							
	Q3	0.784	0.045	***							
	Q4	0.805	0.046	***							
	Q5	0.806	0.045	***							
	Q6	0.80	0.045	***							
	Q7	0.794	0.046	***							
	Q8	0.802	0.046	***							
F2	Q9	0.737			0.616	0.864	0.765	**0.784**			
	Q10	0.809	0.044	***							
	Q11	0.786	0.045	***							
	Q12	0.805	0.045	***							
F3	Q13	0.694			0.584	0.848	0.761	0.699	**0.764**		
	Q14	0.774	0.052	***							
	Q15	0.790	0.054	***							
	Q16	0.794	0.053	***							
F4	Q17	0.701			0.592	0.812	0.679	0.647	0.661	**0.769**	
	Q18	0.801	0.054	***							
	Q19	0.801	0.054	***							
F5	Q20	0.701			0.581	0.805	0.723	0.615	0.659	0.551	**0.762**
	Q21	0.771	0.052	***							
	Q22	0.810	0.055	***							

Bold values indicate the square root of average variance extracted (AVE); AVE = average variance extracted; CR = composite reliability; S.E. = standard error. Source: author composition based on study data.

**Table 7:** The result of the HTMT calculation.

Factors	F5	F4	F3	F2	F1
F5	–				
F4	0.536	–			
F3	0.646	0.645	–		
F2	0.599-	0.638	0.689	–	
F1	0.708	0.670	0.750	0.758	–

Source: author composition based on study data.

### Reliability

The Chinese version of the LMS-N scale demonstrated acceptable internal consistency, with a Cronbach’s *α* coefficient of 0.933 and McDonald’s *ω* of 0.931 for the overall scale. The reliability coefficients for each subscale ranged from 0.811 to 0.925 for Cronbach’s α and from 0.812 to 0.924 for McDonald’s *ω* (Table 8).

**Table 8:** Reliability of Chinese version of LMS-N.

Factors	Cronbach’s alpha	95%CI	McDonald’s *ω*
Management support	0.925	0.920, 0.930	0.924
Visual stock management	0.857	0.847, 0.846	0.857
Work environment layout	0.848	0.837, 0.858	0.848
Preventive reporting system	0.811	0.797, 0.825	0.812
Waste detection	0.823	0.810, 0.836	0.823
Overall	0.933	0.929, 0.937	0.931

Source: author composition based on study data.

Test–retest reliability of the Chinese LMS-N was evaluated using intraclass correlation coefficients (ICC) based on a two-way mixed-effects model with absolute agreement, which is recommended for assessing the consistency of measurements across repeated administrations of the same instrument. A subsample of 220 participants was recruited from the same hospitals included in the main survey, selected to minimize the influence of memory effects while reducing the likelihood of genuine changes in participants’ lean mindset. This interval is commonly recommended in psychological and organizational measurement studies as a balance between these two sources of potential bias (40). ICC values were interpreted according to Koo and Li (41), where ICC < 0.50 indicates poor reliability, 0.50–0.75 moderate reliability, 0.75–0.90 good reliability, and >0.90 excellent reliability. In this study, the ICC was 0.880 (95% CI: 0.847–0.907).

### Measurement invariance

This study employed multi-group confirmatory factor analysis to examine measurement invariance of the Chinese version of the LMS-N across gender, department, and educational attainment groups. The results indicated minimal changes in model fit indices across nested models, with ΔCFI and ΔTLI values below 0.01 and ΔRMSEA below 0.015 (42). Strict invariance was supported across all three grouping variables, indicating equivalence in factor structure, factor loadings, intercepts, and residual variances across groups (Table 9).

**Table 9:** Cross-gender, department and educational attainment measurement invariance of the Chinese version of LMS-N.

Groups	Model	df	*χ*^2^	AIC	BIC	RMSEA	SRMR	CFI	TLI	△*χ*^2^	△df	△RMSEA	△CFI	△TLI
Gender	Configural	398	584.490	136,129.012	136,989.707	0.021	0.019	0.993	0.991	–	–	–	–	–
	Metric	415	600.643	136,111.166	136,875.599	0.021	0.021	0.993	0.992	16.154	17.000	0.000	0.000	0.000
	Scalar	432	625.536	136,102.058	136,770.230	0.021	0.021	0.992	0.992	24.893	17.000	0.000	0.000	0.000
	Strict	454	661.164	136,093.686	136,637.283	0.021	0.021	0.992	0.992	35.628	22.000	0.000	−0.001	0.000
Department	Configural	995	1,318.033	136,380.097	138,531.835	0.028	0.028	0.987	0.985	–	–	–	–	–
	Metric	1,063	1,361.300	136,287.365	138,054.055	0.026	0.031	0.988	0.987	43.267	68.000	−0.002	0.001	0.002
	Scalar	1,131	1,450.715	136,240.780	137,622.422	0.026	0.032	0.987	0.987	89.415	68.000	0.000	−0.001	0.000
	Strict	1,219	1,540.456	136,154.521	137,037.866	0.025	0.032	0.987	0.988	89.741	88.000	−0.001	0.000	0.001
Educational attainment	Configural	597	900.379	136,170.944	137,461.987	0.027	0.023	0.988	0.986	–	–	–	–	–
	Metric	631	941.284	136,143.848	137,242.367	0.026	0.026	0.988	0.987	40.904	34.000	0.000	0.000	0.000
	Scalar	665	979.671	136,114.236	137,020.231	0.026	0.027	0.988	0.987	38.388	34.000	−0.001	0.000	0.001
	Strict	709	1,053.356	136,099.920	136,756.767	0.026	0.027	0.986	0.987	73.685	44.000	0.000	−0.001	0.000

Δ indicates the difference between two values (before and after) Source: author composition based on study data.

## Discussion

This study addressed a gap in the assessment of lean management in Chinese hospital nursing by providing the cross-culturally validated instrument specifically designed for this context. Existing lean assessment tools in healthcare are either adapted from manufacturing-oriented frameworks (13), focused on organisational leadership rather than frontline nursing practice (14), or excessively long for routine clinical application (15). The Chinese version of the LMS-N offers a theoretically grounded, concise, and empirically sound alternative that is basically aligned with the daily realities of nurses working in Chinese tertiary hospitals.

The replication of the original five-factor structure across a substantially larger and more diverse sample than the original Turkish study is theoretically meaningful (5). It suggests that the conceptual domains of lean nursing management—Management Support, Visual Stock Management, Work Environment Layout, Preventive Reporting System, and Waste Detection—are not culturally specific constructs but reflect genuine and transferable features of lean thinking applicable across different healthcare systems. This cross-cultural structural consistency strengthens the theoretical legitimacy of lean management as a universal framework for nursing quality improvement (4, 6), rather than a practice confined to its industrial or Western origins.

The two targeted cultural adaptations made during the translation process—replacing “employees” with “nurses” and substituting “stand-up meeting” with “departmental morning meeting”—proved empirically justified. Rather than diluting the original construct, these changes appear to have enhanced the scale’s semantic precision within the Chinese clinical context, as evidenced by the high expert consensus on content validity and the clean factor loadings observed in both EFA and CFA. This underscores a broader principle in cross-cultural instrument adaptation: fidelity to conceptual equivalence, rather than literal translation, is the more appropriate standard for producing valid measures across cultural settings (24).

Among the five dimensions, Management Support yielded the highest reliability coefficient, which is consistent with the structural characteristics of Chinese hospital systems. In hierarchical, top-down organisations, where nursing workflow reforms require explicit endorsement from administrators, nurses are likely to have more differentiated and strongly held perceptions of management support compared to other lean dimensions. This heightened sensitivity also implies that the Management Support subscale may serve as a particularly responsive indicator for evaluating the impact of leadership-driven lean initiatives—a finding with direct relevance to nurse managers seeking to prioritise entry points for lean implementation (9, 16).

The Preventive Reporting System dimension, which assesses nurses’ proactive identification and reporting of near-miss events, warrants particular clinical attention. Although it demonstrated satisfactory psychometric properties, its reliability estimates were the lowest among the five subscales. This pattern may reflect genuine variability in near-miss reporting culture across departments and institutions in China, where punitive reporting environments or organisational norms around error disclosure can suppress transparent communication (11). Rather than being a measurement weakness, this variability may signal that the Preventive Reporting System dimension is capturing a real and meaningful heterogeneity in safety culture—exactly the kind of information that lean management assessment should surface for targeted quality improvement.

The replication of the original five-factor structure across a substantially larger sample than the original Turkish study provides preliminary evidence supporting the structural stability of the LMS-N within the studied context. It suggests that the conceptual domains of lean nursing management—Management Support, Visual Stock Management, Work Environment Layout, Preventive Reporting System, and Waste Detection—may capture meaningful aspects of nursing practice in Chinese tertiary hospital settings. However, given that the present study was conducted using convenience sampling in tertiary hospitals from a single province in China, these findings should be interpreted as context-specific evidence rather than proof of universal applicability. Further studies involving more diverse geographic regions, hospital levels, and sampling strategies are needed to confirm the generalisability of the scale across broader healthcare settings.

From an applied perspective, the availability of a validated Chinese version of the LMS-N may provide a useful tool for assessing lean management implementation in nursing practice within similar tertiary hospital contexts. The five-dimensional structure offers a framework that may help identify potential areas for quality improvement, including leadership support, workflow organisation, safety reporting practices, and waste reduction behaviours. However, the present findings should not be directly interpreted as evidence for system-wide policy implementation. Instead, they may serve as a reference point for local hospital-level quality improvement initiatives, while broader policy applications would require further validation across different regions and healthcare settings in China.

## Conclusion

This study translated and culturally adapted the Lean Management Scale for Nursing Services in Hospitals (LMS-N) into Chinese and examined its psychometric properties among clinical nurses in tertiary hospitals in one province of China. The results provide preliminary evidence supporting the scale’s reliability and validity, including acceptable content, construct, convergent and discriminant validity, internal consistency, and test–retest reliability. Measurement invariance across gender, department, and educational level was also supported within the study sample.

These findings are broadly consistent with the psychometric performance reported for the original LMS-N developed by Çiğdem et al., which also demonstrated good model fit and acceptable internal consistency across its five-dimension structure (21). This suggests that the core factor structure of the LMS-N translates reasonably well across cultural and healthcare-system contexts. The reliability and validity indices obtained in this study are also comparable to those reported for other recently validated Chinese nursing instruments. For example, a 2026 nurse-specific scale assessing clinical practice guideline implementation factors among Chinese nurses reported strong overall reliability (Cronbach’s *α* = 0.972) and acceptable confirmatory factor analysis fit indices (*χ*^2^/df = 1.956, RMSEA = 0.055, CFI = 0.935, TLI = 0.929) (43). This comparison indicates that the Chinese LMS-N performs at a level consistent with established standards in the nursing instrument validation literature in China, while extending this work into the domain of lean management, an area that has so far lacked a validated, nursing-specific measurement tool in this context.

However, the findings should be interpreted in light of several limitations, including convenience sampling from a single province, reliance on self-report data, lack of criterion validity assessment, and the cross-sectional design. Future research should extend validation to more geographically and institutionally diverse samples, including secondary and primary-level hospitals, and examine the scale’s responsiveness to lean management interventions over time using longitudinal designs.

Overall, the Chinese LMS-N may be useful for evaluating lean management in nursing practice in similar hospital settings. Its sound psychometric properties support its potential value as both a research tool and a practical resource for nurse managers seeking to monitor and advance lean management implementation. Further validation in more diverse populations is needed to confirm its broader applicability and to establish normative benchmarks for clinical and managerial use.

## Limitations and future work

First, the sample was drawn exclusively from tertiary hospitals in Jinzhou, Liaoning Province, limiting generalisability to primary or secondary care settings, rural hospitals, or other regions of China where lean management culture and nurse demographics may differ substantially. Second, common method bias cannot be entirely excluded given the self-report design, and social desirability bias may be particularly relevant in hierarchical Chinese hospital settings. Third, criterion-related validity was not assessed; therefore, although the LMS-N demonstrated satisfactory psychometric performance, the extent to which higher scores reflect actual improvements in nursing quality, patient outcomes, or objective lean implementation remains unclear. Future studies should examine associations with nursing-sensitive quality indicators, patient safety incident rates, and objective lean management metrics. Fourth, convenience sampling may have introduced selection bias, with nurses in hospitals with more established lean programmes potentially overrepresented. Finally, the cross-sectional design precludes evaluation of the scale’s responsiveness to change, limiting its current use for monitoring longitudinal progress or evaluating the effectiveness of quality improvement interventions. Longitudinal studies are needed to determine whether the scale can detect meaningful changes following lean implementation initiatives.

## Data Availability

The raw data supporting the conclusions of this article will be made available by the authors, without undue reservation.

## References

[1] Ugwu CN Ugwu OP-C Alum EU Eze VHU Basajja M Ugwu JN . Sustainable development goals (SDGs) and resilient healthcare systems: addressing medicine and public health challenges in conflict zones. Medicine. (2025) 104:e41535. doi: 10.1097/MD.0000000000041535, 39960902 PMC11835129

[2] Roth C Wensing M Breckner A Mahler C Krug K Berger S. Keeping nurses in nursing: a qualitative study of German nurses’ perceptions of push and pull factors to leave or stay in the profession. BMC Nurs. (2022) 21:48. doi: 10.1186/s12912-022-00822-4, 35193561 PMC8863506

[3] Fernandez R Johnson M Tran DT Miranda C. Models of care in nursing: a systematic review. Int J Evid Based Healthc. (2012) 10:324–37. doi: 10.1111/j.1744-1609.2012.00287.x, 23173657

[4] Teich ST Faddoul FF. Lean management—the Journey from Toyota to healthcare. Rambam Maimonides Med J. (2013) 4:e0007. doi: 10.5041/RMMJ.10107, 23908857 PMC3678835

[5] Kılıç ÇT Öztürk H. Development and psychometric testing of the lean management scale for nursing Services in Hospitals. Int J of Nursing Practice. (2024) 30:e13314. doi: 10.1111/ijn.13314, 39443285 PMC11608920

[6] Ahmed S Hawarna S Alqasmi I Ashrafi DM Rahman MK. Mediating role of lean management on the effects of workforce management and value-added time in private hospitals. Int J Lean Six Sigma. (2023) 14:1035–54. doi: 10.1108/IJLSS-05-2022-0102

[7] Geiger-Brown J Lipscomb J. The health care work environment and adverse health and safety consequences for nurses. Annu Rev Nurs Res. (2010) 28:191–231. doi: 10.1891/0739-6686.28.191, 21639028

[8] Ferraro A Centobelli P Cerchione R Cicco MVD Montella E Raiola E . Implementation of lean practices to reduce healthcare associated infections. Int J Healthc Technol Manag. (2020) 18:51–72. doi: 10.1504/IJHTM.2020.116783

[9] Zhang J Mitchell R Zhao R Li M Wang W. What is successful integration in primary health care: qualitative insights from the Chinese public. Glob Health Action. (2024) 17:2430811. doi: 10.1080/16549716.2024.2430811, 39558840 PMC11578409

[10] Yuan L Cao J Wang D Yu D Liu G Qian Z. Regional disparities and influencing factors of high quality medical resources distribution in China. Int J Equity Health. (2023) 22:8. doi: 10.1186/s12939-023-01825-6, 36627636 PMC9832614

[11] Phillips J Malliaris AP Bakerjian D. Nursing and Patient Safety. (2024).

[12] Sipe MH Kennedy K. Integrating human-centered approaches to align doctor of nursing practice curriculum with real-world practice. Nurse Lead. (2024) 22:298–302. doi: 10.1016/j.mnl.2024.03.009

[13] Machado Guimarães C Crespo de Carvalho J. Assessing lean deployment in healthcare—a critical review and framework. J Enterp Transform. (2014) 4:3–27. doi: 10.1080/19488289.2013.869277

[14] Reponen E Jokela R Blodgett JC Rundall TG Shortell SM Nuutinen M . Validation of the lean healthcare implementation self-assessment instrument (LHISI) in the finnish healthcare context. BMC Health Serv Res. (2021) 21:1289. doi: 10.1186/s12913-021-07322-2, 34852808 PMC8638099

[15] Roszell SS Lynn MR. A measure of lean quality improvement for hospital staff nurses. J Nurs Care Qual. (2016) 31:373–9. doi: 10.1097/NCQ.0000000000000185, 26998578

[16] Chen M Guan Q Zhuang J. Patient-centered lean healthcare management from a humanistic perspective. BMC Health Serv Res. (2024) 24:1261. doi: 10.1186/s12913-024-11755-w, 39427202 PMC11490186

[17] Wang J Lv H Chen M Liu C Ren W Jiang H . A systematic review of lean implementation in hospitals: impact on efficiency, quality, cost, and satisfaction. Int J Health Policy Manag. (2025) 14:8974. doi: 10.34172/ijhpm.8974, 41966206 PMC12573144

[18] Sallam M Allam D Kassem R. Improving efficiency in hospital pharmacy services: an integrated strategy using the OCTAGON-P framework and lean 5S management practices. Cureus. (2024) 16:e56965. doi: 10.7759/cureus.56965, 38665739 PMC11044978

[19] Luo M Yue Y Li W Fang Q. Adaptability research on lean management under medical insurance payment reform: threshold effect of DRG/DIP reform intensity and operational efficiency of traditional chinese medicine hospitals. Front Public Health. (2025) 13:1715476. doi: 10.3389/fpubh.2025.1715476, 41561876 PMC12813153

[20] Chen M Li X Wang A Zhou B. Developing and validating the chinese nursing core competence scale for tertiary hospital nurses based on evolving health care needs in China. BMC Nurs. (2025) 24:728. doi: 10.1186/s12912-025-03384-3, 40598261 PMC12211946

[21] Fu Z Wang Y Zhang L Tan M. Translation and psychometric evaluation of the chinese version of the nursing time management scale. Front Med. (2024) 11:1396625. doi: 10.3389/fmed.2024.1396625, 38799153 PMC11116688

[22] Li C Lin Y Tosun B Wang P Guo HY Ling CR . Psychometric evaluation of the Chinese version of the BENEFITS-CCCSAT based on CTT and IRT: a cross-sectional design translation and validation study. Front Public Health. (2025) 13:1532709. doi: 10.3389/fpubh.2025.1532709 40171423 PMC11960502

[23] Kyriazos TA. Applied psychometrics: sample size and sample power considerations in factor analysis (EFA, CFA) and SEM in general. Psychology. (2018) 9:2207–30. doi: 10.4236/psych.2018.98126

[24] Ding Y Zhang Y Yue S. The psychometric properties of the chinese version of the pain relief motivation scale in patients with neurogenic chronic pain. Pain Manag Nurs: Off J Am Soc Pain Manag Nurses. (2025) 26:e318–24. doi: 10.1016/j.pmn.2024.11.004, 39638738

[25] Li C Meng ZX Lin YB Zhang L. Cross-cultural adaptation and psychometric evaluation of the Chinese version of the sickness presenteeism scale-nurse (C-SPS-N): a cross-sectional study. BMC Nurs. (2025) 24:494. doi: 10.1186/s12912-025-03113-w, 40336046 PMC12057012

[26] Aksah H Johar S Usman IM Ani AIC. Design and implementation of content validity: instrument development for evaluating functional building performance. WSEAS Trans Environ Dev. (2021) 17:973–82. doi: 10.37394/232015.2021.17.90

[27] Cheung GW Cooper-Thomas HD Lau RS Wang LC. Correction to: reporting reliability, convergent and discriminant validity with structural equation modeling: a review and best-practice recommendations. Asia Pac J Manag. (2024) 41:785–7. doi: 10.1007/s10490-023-09880-x

[28] Hair JF Hult GTM Ringle CM Sarstedt M Danks NP Ray S. "Evaluation of reflective measurement models". In: Hair JF Jr Hult GTM Ringle CM Sarstedt M Danks NP Ray S, editors. Partial least Squares Structural Equation Modeling (PLS-SEM) using R: a Workbook. Cham: Springer International Publishing (2021). p. 75–90. doi: 10.1007/978-3-030-80519-7_4

[29] Vandenberg RJ Lance CE. A review and synthesis of the measurement invariance literature: suggestions, practices, and recommendations for organizational research. Organ Res Methods. (2000) 3:4–70. doi: 10.1177/109442810031002

[30] Kong L Lu T Zheng C Zhang H. Psychometric evaluation of the Chinese version of the positive health Behaviours scale for clinical nurses: a cross-sectional translation. BMC Nurs. (2023) 22:296. doi: 10.1186/s12912-023-01453-z, 37653399 PMC10470149

[31] Shao Y Zhang H Zhang X Liang Q Zhang H Zhang F. Chinese version of exercise dependence scale-revised: psychometric analysis and exploration of risk factors. Front Psychol. (2023) 14:1309205. doi: 10.3389/fpsyg.2023.1309205, 38115976 PMC10728468

[32] Li C Lin Y Cheng J Xu D Zhang L. Psychometric properties of the Chinese version of scales of knowledge, attitude, and practice of self-care for patients with arteriovenous fistula: a translation and verification study. Front Public Health. (2025) 13:1588271. doi: 10.3389/fpubh.2025.1588271, 40356843 PMC12066288

[33] Hoseinzadeh E Sharif-Nia H Ashktorab T Ebadi A. Development and psychometric evaluation of nurse’s intention to care for patients with infectious disease scale: an exploratory sequential mixed method study. BMC Nurs. (2024) 23:65. doi: 10.1186/s12912-023-01669-z, 38267951 PMC10807223

[34] Cicolini G Della Pelle C Cerratti F Franza M Flacco ME. Validation of the Italian version of the Stanford Presenteeism scale in nurses. J Nurs Manag. (2016) 24:598–604. doi: 10.1111/jonm.12362, 26935013

[35] Nkansah BK On the Kaiser-Meier-Olkin’s Measure of Sampling Adequacy. (2018).

[36] Yılmaz D Uzelli D Dikmen Y. Psychometrics of the attitude scale towards the use of artificial intelligence Technologies in Nursing. BMC Nurs. (2025) 24:151. doi: 10.1186/s12912-025-02732-7, 39930420 PMC11809082

[37] Ferreira LK Filgueiras Meireles JF de Oliveira Gomes GA Caputo Ferreira ME. Development and psychometric evaluation of a lifestyle evaluation instrument for older adults. Percept Mot Skills. (2023) 130:1901–23. doi: 10.1177/00315125231182173 37286477

[38] Yang Z Chen F Lu Y Zhang H. Psychometric evaluation of medication safety competence scale for clinical nurses. BMC Nurs. (2021) 20:165. doi: 10.1186/s12912-021-00679-z, 34503485 PMC8428106

[39] Yue M Chen Q Liu Y Cheng R Zeng D. Psychometric properties of the Chinese version of the nurses’ attitudes towards communication with the patient scale among Chinese nurses. BMC Nurs. (2024) 23:779. doi: 10.1186/s12912-024-02415-9, 39443989 PMC11515601

[40] Smith KRH. Disability discrimination in employment. Occup Med (Lond). (2010) 60:156–6. doi: 10.1093/occmed/kqp178

[41] Koo TK Li MY. A guideline of selecting and reporting intraclass correlation coefficients for reliability research. J Chiropr Med. (2016) 15:155–63. doi: 10.1016/j.jcm.2016.02.012, 27330520 PMC4913118

[42] Khademi A Wells CS Oliveri ME Villalonga-Olives E. Examining appropriacy of CFI and TLI cutoff value in multiple-group CFA test of measurement invariance to enhance accuracy of test score interpretation. SAGE Open. (2023) 13:21582440231205354. doi: 10.1177/21582440231205354

[43] Liu L Dallakoti N Cui W Li X Ding S Zhang S. Development and validation of a nurse-specific scale to assess influencing factors in clinical practice guideline implementation: an i-PARIHS-based study. J Nurs Manag. (2026) 2026:4584848. doi: 10.1155/jonm/4584848, 41635895 PMC12862181

