# Supplementary material for: Cross-cultural validation of the Chinese version of the lean management scale for nursing services in hospitals
Source: Front Public Health. 2026 Jul 29;14:1895347. doi: 10.3389/fpubh.2026.1895347 (PMC13461817; doi:10.3389/fpubh.2026.1895347)
Supplement: Supplementary file 1 [file Table_1.docx]

Supplementary Material 1

| Age and years of work experience: | | | | | |
| --- | --- | --- | --- | --- | --- |
|  | G | N | Mean | Std. Deviation | Std. Error Mean |
| Age | EFA | 1027 | 39.18 | 9.492 | .296 |
|  | CFA | 1100 | 38.66 | 9.566 | .288 |
| years of work experience | EFA | 1027 | 15.60 | 8.498 | .265 |
|  | CFA | 1100 | 15.54 | 8.638 | .260 |

|  | | t-test for Equality of Means | | | | | | |
| --- | --- | --- | --- | --- | --- | --- | --- | --- |
|  |  | t | df | Sig. (2-tailed) | Mean Difference | Std. Error Difference | 95% Confidence Interval of the Difference | |
|  |  |  |  |  |  |  | Lower | Upper |
| Age | Equal variances assumed | 1.254 | 2125 | .210 | .519 | .414 | -.292 | 1.329 |
|  | Equal variances not assumed | 1.254 | 2117.139 | .210 | .519 | .413 | -.292 | 1.329 |
| years of work experience | Equal variances assumed | .181 | 2125 | .856 | .067 | .372 | -.662 | .797 |
|  | Equal variances not assumed | .181 | 2119.192 | .856 | .067 | .372 | -.662 | .796 |

Gender:

|  | | | G | | Total |
| --- | --- | --- | --- | --- | --- |
|  |  |  | CFA | EFA |  |
| Gender | males | Count | 315 | 323 | 638 |
|  |  | Expected Count | 329.9 | 308.1 | 638.0 |
|  | females | Count | 785 | 704 | 1489 |
|  |  | Expected Count | 770.1 | 718.9 | 1489.0 |
| Total | | Count | 1100 | 1027 | 2127 |
|  |  | Expected Count | 1100.0 | 1027.0 | 2127.0 |

| **Chi-Square Tests** | | | | | |
| --- | --- | --- | --- | --- | --- |
|  | Value | df | Asymptotic Significance (2-sided) | Exact Sig. (2-sided) | Exact Sig. (1-sided) |
| Pearson Chi-Square | 2.004^a^ | 1 | .157 |  |  |
| Continuity Correction^b^ | 1.872 | 1 | .171 |  |  |
| Likelihood Ratio | 2.003 | 1 | .157 |  |  |
| Fisher's Exact Test |  |  |  | .170 | .086 |
| Linear-by-Linear Association | 2.003 | 1 | .157 |  |  |
| N of Valid Cases | 2127 |  |  |  |  |
| a. 0 cells (0.0%) have expected count less than 5. The minimum expected count is 308.05. | | | | | |
| b. Computed only for a 2x2 table | | | | | |

| Educational attainment: | | | | | |
| --- | --- | --- | --- | --- | --- |
|  | | | G | | Total |
|  |  |  | CFA | EFA |  |
| Educational attainment | professional training college | Count | 117 | 95 | 212 |
|  |  | Expected Count | 109.6 | 102.4 | 212.0 |
|  | undergraduate | Count | 707 | 667 | 1374 |
|  |  | Expected Count | 710.6 | 663.4 | 1374.0 |
|  | postgraduates | Count | 276 | 265 | 541 |
|  |  | Expected Count | 279.8 | 261.2 | 541.0 |
| Total | | Count | 1100 | 1027 | 2127 |
|  |  | Expected Count | 1100.0 | 1027.0 | 2127.0 |

| **Chi-Square Tests** | | | |
| --- | --- | --- | --- |
|  | Value | df | Asymptotic Significance (2-sided) |
| Pearson Chi-Square | 1.167^a^ | 2 | .558 |
| Likelihood Ratio | 1.170 | 2 | .557 |
| Linear-by-Linear Association | .708 | 1 | .400 |
| N of Valid Cases | 2127 |  |  |
| a. 0 cells (0.0%) have expected count less than 5. The minimum expected count is 102.36. | | | |

| Department: | | | | | |
| --- | --- | --- | --- | --- | --- |
|  | | | G | | Total |
|  |  |  | CFE | EFA |  |
| Department | internal | Count | 323 | 298 | 621 |
|  |  | Expected Count | 321.2 | 299.8 | 621.0 |
|  | surgery | Count | 298 | 286 | 584 |
|  |  | Expected Count | 302.0 | 282.0 | 584.0 |
|  | emergency | Count | 248 | 233 | 481 |
|  |  | Expected Count | 248.8 | 232.2 | 481.0 |
|  | ICU | Count | 163 | 156 | 319 |
|  |  | Expected Count | 165.0 | 154.0 | 319.0 |
|  | others | Count | 68 | 54 | 122 |
|  |  | Expected Count | 63.1 | 58.9 | 122.0 |
| Total | | Count | 1100 | 1027 | 2127 |
|  |  | Expected Count | 1100.0 | 1027.0 | 2127.0 |

| **Chi-Square Tests** | | | |
| --- | --- | --- | --- |
|  | Value | df | Asymptotic Significance (2-sided) |
| Pearson Chi-Square | .977^a^ | 4 | .913 |
| Likelihood Ratio | .979 | 4 | .913 |
| Linear-by-Linear Association | .086 | 1 | .770 |
| N of Valid Cases | 2127 |  |  |
| a. 0 cells (0.0%) have expected count less than 5. The minimum expected count is 58.91. | | | |

| Professional title: | | | | | |
| --- | --- | --- | --- | --- | --- |
|  | | | G | | Total |
|  |  |  | CFA | EFA |  |
| Professional title | nurse | Count | 323 | 298 | 621 |
|  |  | Expected Count | 321.2 | 299.8 | 621.0 |
|  | senior nurse | Count | 298 | 286 | 584 |
|  |  | Expected Count | 302.0 | 282.0 | 584.0 |
|  | supervisor nurse | Count | 248 | 233 | 481 |
|  |  | Expected Count | 248.8 | 232.2 | 481.0 |
|  | associate chief nurse | Count | 163 | 156 | 319 |
|  |  | Expected Count | 165.0 | 154.0 | 319.0 |
|  | chief nurse | Count | 68 | 54 | 122 |
|  |  | Expected Count | 63.1 | 58.9 | 122.0 |
| Total | | Count | 1100 | 1027 | 2127 |
|  |  | Expected Count | 1100.0 | 1027.0 | 2127.0 |

| **Chi-Square Tests** | | | |
| --- | --- | --- | --- |
|  | Value | df | Asymptotic Significance (2-sided) |
| Pearson Chi-Square | .977^a^ | 4 | .913 |
| Likelihood Ratio | .979 | 4 | .913 |
| Linear-by-Linear Association | .086 | 1 | .770 |
| N of Valid Cases | 2127 |  |  |
| a. 0 cells (0.0%) have expected count less than 5. The minimum expected count is 58.91. | | | |

| marital status: | | | | | |
| --- | --- | --- | --- | --- | --- |
|  | | | G | | Total |
|  |  |  | 1 | 2 |  |
| marital status | married | Count | 659 | 625 | 1284 |
|  |  | Expected Count | 664.0 | 620.0 | 1284.0 |
|  | unmarried | Count | 376 | 342 | 718 |
|  |  | Expected Count | 371.3 | 346.7 | 718.0 |
|  | others | Count | 65 | 60 | 125 |
|  |  | Expected Count | 64.6 | 60.4 | 125.0 |
| Total | | Count | 1100 | 1027 | 2127 |
|  |  | Expected Count | 1100.0 | 1027.0 | 2127.0 |

| **Chi-Square Tests** | | | |
| --- | --- | --- | --- |
|  | Value | df | Asymptotic Significance (2-sided) |
| Pearson Chi-Square | .205^a^ | 2 | .902 |
| Likelihood Ratio | .205 | 2 | .902 |
| Linear-by-Linear Association | .150 | 1 | .699 |
| N of Valid Cases | 2127 |  |  |
| a. 0 cells (0.0%) have expected count less than 5. The minimum expected count is 60.35. | | | |
